# Redundant electrostatic interactions between GATOR1 and the Rag GTPase heterodimer drive efficient amino acid sensing in human cells

**DOI:** 10.1016/j.jbc.2023.104880

**Published:** 2023-06-01

**Authors:** Dylan D. Doxsey, Steven D. Tettoni, Shawn B. Egri, Kuang Shen

**Affiliations:** 1 Program in Molecular Medicine, University of Massachusetts Chan Medical School, Worcester, Massachusetts, USA; 2 Department of Biochemistry & Molecular Pharmacology, University of Massachusetts Chan Medical School, Worcester, Massachusetts, USA

**Keywords:** mTOR complex 1 (mTORC1), Rag GTPase, GATOR1, metabolism, GTPase activating protein

## Abstract

Cells need to coordinate nutrient availability with their growth and proliferation. In eukaryotic cells, this coordination is mediated by the mechanistic target of the rapamycin complex 1 (mTORC1) pathway. mTORC1 activation is regulated by two GTPase units, the Rag GTPase heterodimer and the Rheb GTPase. The RagA-RagC heterodimer controls the subcellular localization of mTORC1, and its nucleotide loading states are strictly controlled by upstream regulators including amino acid sensors. A critical negative regulator of the Rag GTPase heterodimer is GATOR1. In the absence of amino acids, GATOR1 stimulates GTP hydrolysis by the RagA subunit to turn off mTORC1 signaling. Despite the enzymatic specificity of GATOR1 to RagA, a recent cryo-EM structural model of the human GATOR1-Rag-Ragulator complex reveals an unexpected interface between Depdc5, a subunit of GATOR1, and RagC. Currently, there is no functional characterization of this interface, nor do we know its biological relevance. Here, combining structure-function analysis, enzymatic kinetic measurements, and cell-based signaling assays, we identified a critical electrostatic interaction between Depdc5 and RagC. This interaction is mediated by the positively charged Arg-1407 residue on Depdc5 and a patch of negatively charged residues on the lateral side of RagC. Abrogating this interaction impairs the GAP activity of GATOR1 and cellular response to amino acid withdrawal. Our results reveal how GATOR1 coordinates the nucleotide loading states of the Rag GTPase heterodimer, and thus precisely controls cellular behavior in the absence of amino acids.

Cells need to ensure the abundance of nutrients, such as glucose and amino acids in their local environment before they can commit to growing (1, 2, 3, 4, 5). In contrast, when they are under starvation conditions, they stop growing to conserve resources (6, 7, 8). In eukaryotic cells, the coordination of cell growth with the availability of nutrients is controlled by the mechanistic Target of Rapamycin Complex 1 (mTORC1) (1, 2, 3, 4, 5, 9, 10, 11). mTORC1 is a serine/threonine kinase complex that receives signals from upstream sensors and phosphorylates downstream targets to match the current state of the cell (4, 11, 12, 13, 14). When mTORC1 is activated, it promotes anabolic processes such as protein and lipid synthesis and ribosome biogenesis (15, 16, 17, 18, 19, 20, 21). When mTORC1 is inactivated, it promotes catabolic processes such as autophagy (7).

In human cells, mTORC1 activation requires two GTPase units, the Rag GTPase heterodimer and the Rheb GTPase. When amino acid levels are high, the Rag GTPase heterodimer is activated by its upstream regulators, causing the RagA subunit to be bound to GTP and the RagC subunit to be bound to GDP (22, 23). Under this nucleotide loading configuration, the Rag GTPase heterodimer binds to the Raptor subunit of mTORC1 to recruit it to the lysosomal surface (24, 25, 26, 27). Here, if growth factor signals are also presented, the Rheb GTPase switches to the GTP-bound, active conformation that triggers internal conformational changes within mTORC1 and turns on its kinase activity (28, 29, 30, 31). This coincidental detector ensures that cells will grow only when both amino acids and growth factor signals are present.

The biological activity of the Rag GTPase heterodimer is determined by the nucleotide loading states of both Rag subunits. The active and inactivated states of the Rag GTPase heterodimer require the two subunits to be occupied by “opposite” nucleotides (GTP *versus* GDP). Intrinsically, the two subunits use unique mechanisms of intersubunit communication to coordinate their nucleotide loading states. This system prevents both subunits from binding to the same nucleotide, thus locking the heterodimer in either the active (^GTP^RagA-RagC^GDP^) or the inactive (^GDP^RagA-RagC^GTP^) state (22, 23, 32). The active and inactive configurations of the Rag GTPase heterodimer are kinetically stable, which ensures a steady output toward mTORC1 and also poses a kinetic barrier when nutrient levels change and the cells must respond. Multiple protein complexes such as GATOR1 and FLCN-FNIP2 (33, 34, 35, 36, 37) serve as GTPase activating proteins (GAPs) for the Rag subunits, in order to lower the kinetic barrier for state switching. Other upstream regulators include guanine nucleotide exchange factors (GEFs), such as SLC38A9 and Ragulator (38, 39).

GATOR1 is a major negative regulator of the mTORC1 pathway. It is comprised of three subunits, nitrogen permease-like protein 2 (Nprl2), nitrogen permease-like protein 3 (Nprl3), and DEP-domain containing protein 5 (Depdc5) (33, 34). GATOR1 is a GAP for RagA that stimulates GTP hydrolysis to convert it to the GDP-bound state. The mechanism by which GATOR1 regulates the Rag GTPase heterodimer has been suggested previously, and multiple structural models have been proposed (40). Recently, we found that GATOR1 binds to the Rag GTPases in two distinct, non-exclusive modes: a GAP mode and an inhibitory mode (41). When GATOR1 binds the Rag GTPase heterodimer in the inhibitory mode, it has a high binding affinity but low catalytic efficiency. When GATOR1 binds the Rag GTPase heterodimer in the GAP mode, it has a higher catalytic efficiency which is balanced with a lower affinity. It is proposed that the inhibitory binding mode is responsible for preventing the over-inactivation of the Rag GTPases by a small pool of GATOR1 (40).

The GAP activity of GATOR1 is specific to the RagA subunit. However, in our recent cryo-electron microscopy (cryo-EM) structural model, GATOR1 makes extensive contacts with both RagA and RagC subunits at two interfaces in the GAP mode: Nprl2-Nprl3 contacts RagA and Arg-78 of Nprl2 carries out the enzymatic function (42), while Depdc5 interacts with RagC, forming an additional, auxiliary interface (Fig. 1*A*). By forming this additional contact, GATOR1 pulls RagC away from RagA and breaks the intersubunit communication that locks up the Rag GTPase heterodimer, allowing RagC to bind to GTP (41). As a consequence, after RagA hydrolyzes the bound GTP upon stimulation by Nprl2-Nprl3, the Rag GTPase heterodimer naturally adopts the inactivated state, ^GDP^RagA-RagC^GTP^. This prevents promiscuity that would be caused by direct hydrolysis, leading to the dual GDP-loaded state. Currently, the biochemical properties and the biological relevance of the auxiliary interface remain elusive.

**Figure 1:**
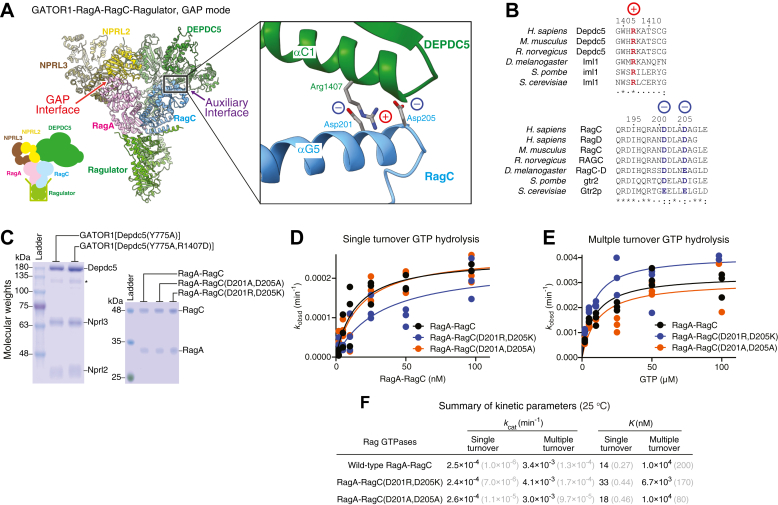
**Identification of structural bases that mediate the auxiliary interaction.***A*, cryo-EM structural model of GATOR1 bound to RagA-RagC-Ragulator in the GAP mode (PDB: 7T3C). Zooming in on the auxiliary interface between Depdc5 and RagC, a patch of charged residues could be responsible for the interaction between GATOR1 and the Rag GTPases. Highlighted is residue Arg-1407 of Depdc5 and two aspartate residues (Asp-201 and Asp-205) on RagC. *B*, sequence alignments showing the evolutionary conservation of the charged residues at the auxiliary interface. *C*, Coomassie-stained gels of the purified GATOR1 complex with Depdc5(R1407D) mutation and of the Rag GTPases with RagC mutations. *Star* denotes a commonly observed Depdc5 degradation product during protein purification. *D*, single turnover GTP hydrolysis by wild-type Rag GTPases (*black*), RagA-RagC(D201A, D205A) (*orange*), and RagA-RagC(D201R, D205K) (*blue*). Assays were performed three times with data points from each assay and a representative fitting curve shown. Both mutants behave similarly to wild-type Rag GTPases. *E*, multiple turnover GTP hydrolysis by wild-type Rag GTPases (*black*), RagA-RagC(D201A, D205A) (*orange*), and RagA-RagC(D201R, D205K) (*blue*). Assays were performed three times with data points from each assay and a representative fitting curve shown. Both mutants behave similarly to wild-type Rag GTPases. *F*, summary of kinetic parameters of intrinsic GTP hydrolysis by the Rag GTPases. The gray numbers in parentheses are SEM calculated from three independent experiments.

In this study, we carried out a structure–function analysis on the auxiliary interface between RagC and Depdc5. We identified a critical electrostatic interaction between the two, mediated by the Arg-1407 residue on Depdc5 and a patch of negatively charged residues on the αG5 helix of RagC. We found that this interaction is essential for GATOR1 to properly carry out its GAP function, with the loss of this interaction resulting in a much lower GAP efficiency. Finally, we showed that Depdc5–RagC interaction is essential for proper mTORC1 response in cells. Our results demonstrate how GATOR1 controls cellular behavior in the absence of amino acids.

## Results and discussion

### Identification of the structural basis for the Depdc5–RagC interaction

To investigate the molecular basis underlying the auxiliary interface, we carefully examined a previously resolved cryo-EM structural model (PDB: 7T3C) (41), in which the nucleotide-binding domain (NBD) of RagC contacts the C-terminal domain (CTD) of the Depdc5 subunit of GATOR1 (Fig. 1*A*). In particular, we observed a strong Coulomb density of a positively-charged arginine residue, Arg-1407 of Depdc5, near a patch of negatively-charged residues, Asp-201 and Asp-205, on the αG5 helix of RagC (Fig. 1*A*). These charged residues are within 3 to 5 Å of each other, which allows for electrostatic interactions to form. Moreover, these residues are evolutionarily conserved in charge (Fig. 1*B*). This suggests that Arg-1407 of Depdc5 and Asp-201 and Asp-205 of RagC might be candidates that mediate the GATOR1–RagC auxiliary interaction.

We designed a series of point mutants, aiming to specifically disrupt the potential interaction. We either neutralized the charges of these residues by mutating them to an alanine residue (R1407A on Depdc5, or D201A/D205A on RagC) or reversed the charges (R1407D on Depdc5, or D201R/D205K on RagC). For the GATOR1 mutants, after gel-filtration purification, all three subunits co-eluted in the same fraction, as seen on a Coomassie-stained gel (Fig. 1*C*), suggesting that the mutations do not affect the integrity of the GATOR1 complex. For the Rag mutants, we expressed them in *Escherichia coli* following a previously published protocol (43). This yielded intact Rag GTPase heterodimers that are suitable for measuring their GTP hydrolysis rates (Fig. 1*C*). As a control, we tested their intrinsic hydrolysis rates using a previously established method (32, 43). Under both single- and multiple-turnover conditions, these mutants behave similarly to wild-type Rag GTPases and have minimal effects in GTP hydrolysis (Fig. 1, *D*–*F*), suggesting any defects that we might see in a GATOR1-stimulated assay (see below) are not due to a non-specific disruption of the folding and architecture of the Rag GTPases.

### Arg-1407 of Depdc5 and Asp-201/Asp-205 of RagC mediate critical interactions in GATOR1-stimulated GTP hydrolysis reactions

To investigate how disrupting the auxiliary interface may affect the stimulatory effect of GATOR1, we first measured and compared the Michaelis-Menten kinetics using wild-type and mutant GATOR1. In order to eliminate the competition from binding to the inhibitory mode, we used the GATOR1[Depdc5(Y775A)] as our “wild-type” background, as this mutant specifically traps the Rag GTPases in the GAP mode by disrupting the inhibitory binding site (40).

**Figure 2:**
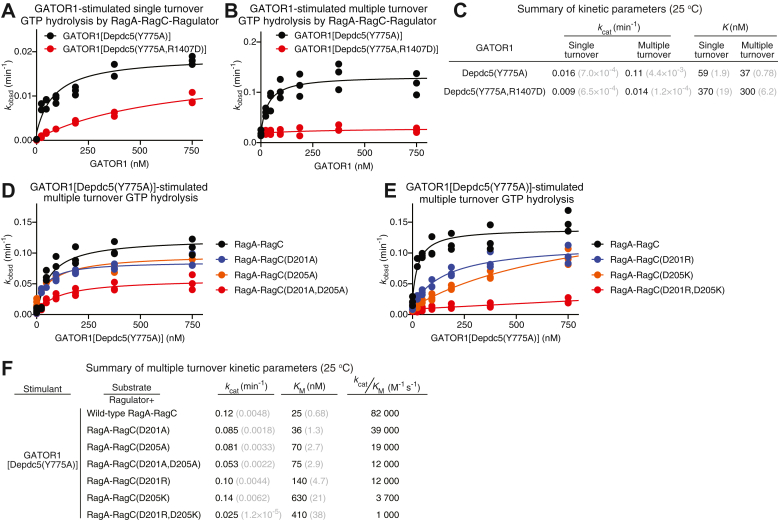
**Depdc5-RagC interaction regulates multiple turnover GTP hydrolysis of the Rag GTPases.***A*, single turnover GTP hydrolysis of the Rag GTPase heterodimer, stimulated by GATOR1[Depdc5(Y775A)] (*black*) or GATOR1[Depdc5(Y775A, R1407D)] (*red*). *B*, multiple turnover GTP hydrolysis of the Rag GTPase heterodimer, stimulated by GATOR1[Depdc5(Y775A)] (*black*) or GATOR1[Depdc5(Y775A,R1407D)] (*red*). *C*, summary of single and multiple turnover GTP hydrolysis kinetics in panels A and B. *Gray numbers* in parentheses are SEM calculated from three independent experiments. *D*, GATOR1[Depdc5(Y775A)]-stimulated multiple turnover GTP hydrolysis of the Rag GTPase heterodimers containing charge-neutralizing mutations. *E*, GATOR1[Depdc5(Y775A)]-stimulated multipleturnover GTP hydrolysis of the Rag GTPase heterodimers containing charge-reversal mutations. *F*, summary of GATOR1[Depdc5(Y775A)]-stimulated multiple turnover GTP hydrolysis kinetics in panels D and E. *Gray numbers* in parentheses are SEM calculated from three independent experiments.

As a control experiment, we validated our GATOR1 construct using a single turnover reaction. Here, the Rag GTPase heterodimer is singly loaded with GTP, with the other subunit unoccupied. Therefore, only two species, ^GTP^RagA-RagC and RagA-RagC^GTP^, exist in the reaction, and the only one that GATOR1 can bind to and stimulate is ^GTP^RagA-RagC. In this scenario, RagC is not loaded with GTP, so it does not have the optimal nucleotide loading configuration to allow for the formation of the auxiliary interface (41). The only binding site available to the Rag GTPases and GATOR1 in the GAP mode is at the GAP interface. When we carried out the stimulatory hydrolysis reactions, the catalytic rate (*k*_cat_) of GATOR1[Depdc5(Y775A)] is 0.016 min^−1^ and that of GATOR1[Depdc5(Y775A, R1407D)] is 0.009 min^−1^ (Fig. 2, *A* and *C*), which are within two-fold of one another. As we hypothesized that the auxiliary interface plays a minor role in the single turnover hydrolysis case scenario, the slight two-fold defect matches our expectation and further confirms the architectural integrity of the GATOR1 complex carrying the R1407D mutation, as well as the functional integrity of its GAP interface.

We then tested the effect of the R1407D mutation on the formation of the auxiliary interface by carrying out the stimulatory GTP hydrolysis reaction using a multiple turnover setup. Here, a saturating concentration of GTP was included so that the Rag GTPase heterodimer is dual-loaded with GTP (^GTP^RagA-RagC^GTP^). In this scenario, the auxiliary interface could readily form (41), and we can then investigate the effect of the R1407D mutation. We observed a strong defect here. When GATOR1[Depdc5(Y775A)] serves as the stimulant, the catalytic rate (*k*_cat_) is 0.11 min^−1^, while the R1407D mutation causes an eight-fold decrease in *k*_cat_ (Fig. 2, *B* and *C*). Moreover, the R1407D mutation introduces an 8-fold increase in *K*_M_, suggesting a weakened binding (Fig. 2, *B* and *C*). We calculated the catalytic efficiency (*k*_cat_/*K*_M_), which represents the effective association rate (32). The overall effect of the R1407D mutation on *k*_cat_/*K*_M_ is a 64-fold decrease, strongly suggesting that the Arg-1407 residue plays a critical role in the formation of the auxiliary interface in the GATOR1-stimulated GTP hydrolysis reaction.

For potential interacting counterparts on RagC, we sought synergistic effects on the Asp-201 and Asp-205 residues. We carried out a similar set of multiple turnover GTP hydrolysis reactions (Fig. 2, *D* and *E*). When we neutralized the charges by mutating individual aspartic acid residues to an alanine residue, we observed a mild defect in the binding between GATOR1 and RagC, as the *k*_cat_/*K*_M_ value decreases by two- and fourfold for D201A and D205A, respectively (Fig. 2*D*, blue and orange lines, and Fig. 2*F*). However, when we introduced a double alanine mutant, we observed a further reduction in the *k*_cat_/*K*_M_ value of ∼sevenfold (Fig. 2*D*, red line, and Fig. 2*F*). These results suggest that the two negatively charged aspartic acid residues form partially redundant interactions with the Arg-1407 residue on Depdc5, as individual substitution cannot completely abrogate the interaction to the baseline level.

Given the nature of the interaction formed between oppositely charged residues, we reasoned that a charge-reversal mutation may induce a more dramatic effect as we change the attraction force to repulsion. Indeed, with a single charge-reversal mutation on RagC (D201R or D205K), the catalytic efficiency (*k*_cat_/*K*_M_) drops by seven- and 22-fold, respectively (Fig. 2*E*, blue and orange lines, and Fig. 2*F*). Moreover, when we reversed the charges on both aspartic acid residues, we observed an 80-fold decrease in the *k*_cat_/*K*_M_ value (Fig. 2*E*, red line, and Fig. 2*F*). These results suggest that reversing the charges which generate a repulsive force at the auxiliary interface leads to a strong binding defect in the GATOR1-stimulated GTP hydrolysis reaction.

### Depdc5-RagC interaction is electrostatic

**Figure 3:**
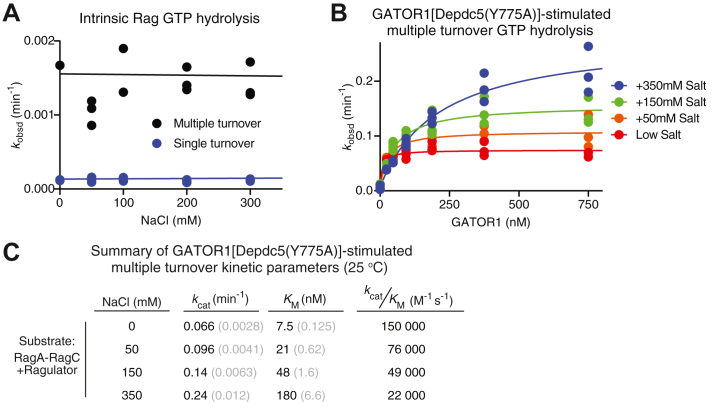
**Depdc5-RagC interaction at the auxiliary interface is electrostatic.***A*, single- and multiple-turnover of GTP by the Rag GTPases alone in the presence of increasing concentrations of salt. *B*, multiple turnover GTP hydrolysis by the Rag GTPases, stimulated by GATOR1[Depdc5(Y775A)] under increasing salt concentrations. *C*, summary of multiple turnover GTP hydrolysis kinetics in panel B. *Gray numbers* in parentheses are SEM calculated from three independent experiments.

As the Depdc5-RagC interaction is mediated by positively- and negatively-charged residues, we hypothesized that it may be electrostatic. If this were the case, increasing the ionic strength of the reaction buffer would weaken the auxiliary interface. To test this hypothesis, we supplemented the reaction buffer with increasing concentrations of sodium chloride (NaCl) and carried out the stimulated GTP hydrolysis reaction as above. As a negative control, we performed single- and multiple-turnover assays with the Rag GTPases alone at increasing concentrations of NaCl (Fig. 3*A*). We did not see any appreciable difference between the conditions, implying that salt does not affect the intrinsic GTP hydrolysis by the Rag GTPases. We then performed the GATOR1[Depdc5(Y775A)]-stimulated multiple turnover assay in various salt concentrations and observed a steady decrease in *k*_cat_/*K*_M_ values as salt concentration increased (Fig. 3, *B* and *C*). This suggests a weakened interaction and supports our hypothesis that the Depdc5-RagC interaction is electrostatic.

### The physiological role of the auxiliary interface in amino acid sensing

**Figure 4:**
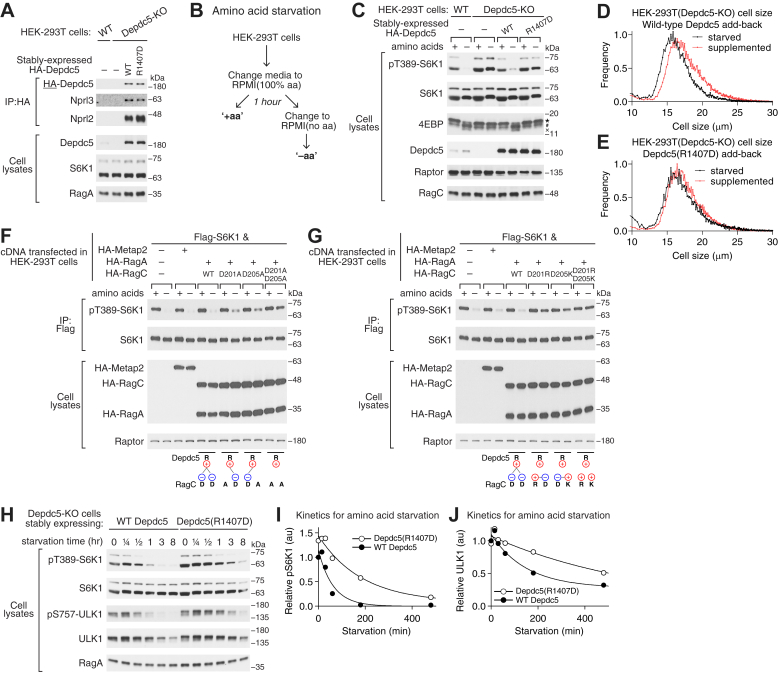
**Disrupting the electrostatic interaction abrogates amino acid signaling in HEK-293T cells.***A*, generation of stable cell lines expressing wild-type Depdc5 or Depdc5(R1407D). Endogenous Depdc5 was first knocked out in HEK-293T cells using CRISPR-Cas9, and HA-tagged wild-type Depdc5 or Depdc5(R1407D) was then stably expressed in the knockout line. Co-immunoprecipitation experiment shows comparable GATOR1 complex formation between wild-type Depdc5 or Depdc5(R1407D) with Nprl2 and Nprl3 (lanes 3 and 4). *B*, amino acid starvation experiment to monitor mTORC1 signaling when cells are deprived of amino acids. *C*, Depdc5(R1407D) fails to restore cellular response to amino acids deprivation in Depdc5-knockout cell line. *Asterisk* denotes hyperphosphorylated 4EBP. *Cross* denotes unphosphorylated 4EBP, which only appears when mTORC1 activity is turned off (lanes 2 and 6). *D*, cell size analysis of HEK-293T cells expressing wild-type Depdc5 under starvation (*black*) or fed (*red*) conditions. The average size of cells under starvation condition is 17.1 μm, while that under fed condition is 18.5 μm. *E*, cell size analysis of HEK-293T cells expressing Depdc5(R1407D) under starvation (*black*) or fed (*red*) conditions. The average size of cells under starvation condition is 18.0 μm, while that under fed condition is 18.3 μm. *F*, amino acid starvation experiment in HEK-293T cells expressing charge-neutralizing RagC mutants. Cellular response to amino acid deprivation was blunted. *G*, amino acid starvation experiment in HEK-293T cells expressing charge-reversal RagC mutants. Cellular response to amino acid deprivation was abrogated. *H*, long-term starvation assay to monitor autophagy mediated by wild-type GATOR1 or GATOR1[Depdc5(R1407D)]. Depdc5(R1407D) severely delays response to amino acid deprivation. *I*, quantification of S6K1 phosphorylation in response to amino acid deprivation. *J*, quantification of ULK1 abundance in response to amino acid deprivation.

To probe the physiological role of the electrostatic interaction and its relevance in amino acid sensing, we need cell lines in which this interaction is specifically impaired. We first used CRISPR-Cas9 to generate a Depdc5-knockout HEK-293T cell line. Western blotting using the Depdc5 antibody confirms the complete knockout (Fig. 4*A*, cell lysates). As shown below, this cell line becomes desensitized to amino acid withdrawal, suggesting a defect in the GATOR1 function. We then stably expressed wild-type or R1407D Depdc5 in the knockout cell line. Expression levels of Depdc5 within the two cell lines are comparable to one another (Fig. 4*A*, cell lysates), as well as their interaction with Nprl2 and Nprl3, suggesting the integrity of the GATOR1 complex is maintained (Fig. 4*A*, IP).

With the stable lines in hand, we tested their response to amino acid withdrawal by depleting the amino acids from the culturing media (Fig. 4*B*). For wild-type cells, 1 h treatment in amino acid-depleted media caused complete inactivation of mTORC1, as the phosphorylation signal of its downstream target, pThr389 on S6K1, disappeared (Fig. 4*C*, lanes 1 and 2). In contrast, for Depdc5-knockout cells, this response is blunted - the cells maintained a high level of phosphorylation on Thr389 of S6K1 even in the absence of amino acids (Fig. 4*C*, lanes 3 and 4), suggesting a negative regulator of the mTORC1 pathway, here GATOR1, failed to carry out its normal function. While expression of wild-type Depdc5 restored the sensitivity to amino acids (Fig. 4*C*, lanes 5 and 6) and normal cell size response to amino acid withdrawal (Fig. 4*D*), expression of Depdc5(R1407D) completely failed to do so (Fig. 4*C*, lanes 7 and 8, and Fig. 4*E*). We also probed the response of another mTORC1 substrate, 4EBP, and observed a similar trend based on its laddering pattern: in the absence of amino acids, hyperphosphorylated 4EBP (asterisk) maintains in the cell line expressing Depdc5(R1407D), while disappears in the wild-type cell lines. These results suggest that the electrostatic interaction between Depdc5 and RagC is necessary for GATOR1 to turn off the mTORC1 pathway in the absence of amino acids.

To test the synergistic effect between Arg-1407 of Depdc5 and the negatively charged residues on RagC, we performed a similar set of experiments using RagC mutants. In wild-type HEK-293T cells, we transfected cDNA expressing either wild-type RagC or charge-neutralizing mutants (D201A and/or D205A) and tested their response to amino acid withdrawal by monitoring the phosphorylation on Thr389 of S6K1 (Fig. 4*F*). When we expressed wild-type Rag GTPases in HEK-293T cells, we did not observe any changes in the cellular response to amino acid deprivation (Fig. 4*F*, lanes 1–6). However, when RagC(D201A) or RagC(D205A) was expressed, we observed a slight elevation of the phosphorylation signal in the absence of amino acid (Fig. 4*F*, lanes 8 and 10), suggesting a partial defect of GATOR1 function in turning off mTORC1 signaling. This defect is relatively mild, which is consistent with the mild reduction in *k*_cat_/*K*_M_ values in stimulated GTP hydrolysis assay *in vitro* (cf. Fig. 2*D*). When we expressed the double alanine mutant in cells (Fig. 4*F*, lanes 11 and 12), we observed a much greater defect, which is also consistent with our previous results (cf. Fig. 2*D*).

To further test our hypothesis, we introduced charge-reversal RagC mutants in HEK-293T cells (Fig. 4*G*). When we expressed single charge-reversal mutants (D201R or D205K), we observed a more severe defect in turning off mTORC1 signaling than the charge-neutralizing mutants (Fig. 4*G*, lanes 7–10). Moreover, the double charge-reversal mutant (D201R/D205K) completely loses sensitivity to amino acid deprivation (Fig. 4*G*, lanes 11 and 12), as the phosphorylation level of Thr389 of S6K1 remains almost identical to the condition where amino acids are well supplemented. This is fully consistent with the *in vitro* results of our GTP hydrolysis experiments, suggesting that the negatively charged residues on RagC are necessary for GATOR1 to carry out its normal function during amino acid deprivation.

mTORC1 is responsible for regulating autophagy in the absence of nutrients. We, therefore, proposed that the auxiliary interaction between GATOR1 and RagC plays a role in initiating autophagy. To test this hypothesis, we used the stable cell lines in a long-term starvation experiment (Fig. 4*H*). Here, we incubated the cells in the absence of amino acids for a prolonged time (∼8 h), and checked the kinetics of a protein marker for autophagy, ULK1. Upon initiation of autophagy, the ULK1 protein level is downregulated by an E3 ligase, NEDD4L (44, 45), which provides a sensitive measure to quantify the autophagy process. As a control experiment, we first probed the phosphorylation level of the Thr389 residue on S6K1 and found that its decrease in the cell line that stably expresses Depdc5(R1407D) is blunted in comparison to the cell line that stably expresses wild-type Depdc5 (Fig. 4*H*, quantified in Fig. 4*I*), which is consistent with our results above. When probing the protein level of ULK1, we observed that the degradation of ULK1 in the Depdc5(R1407D)-expressing cell line is also severely delayed (Fig. 4*H*, quantified in Fig. 4*J*), suggesting the auxiliary interaction between Depdc5 and RagC is necessary for efficient initiation of autophagy under nutrient-deprived conditions. At late time points, autophagy is eventually initiated in the mutant cell line, suggesting parallel pathways, such as GCN2, may kick in and act independently of mTORC1 (46, 47). These mTORC1-independent pathways activate autophagy and may feed back, causing a loss in the mTORC1-dependent markers, such as S6K1 phosphorylation. In general, these signaling experiments in cells corroborate our *in vitro* result and suggest a functional role of the electrostatic interaction between Depdc5 and RagC in transmitting amino acid signals.

### Summary

Amino acid signaling is essential for coordinating cell growth with nutrient levels. Amino acid sensors upstream of the GATOR complexes transmit the signals toward the Rag GTPase heterodimer to control the activity of mTORC1. GATOR1 is a GAP for the Rag GTPase heterodimer, which is an important negative regulator of mTORC1. Despite the enzymatic specificity of GATOR1 to RagA, a recent cryo-EM structural model revealed unexpected interactions between Depdc5 and RagC when GATOR1 carries out its GAP activity. In this study, we identified redundant electrostatic interactions between RagC and Depdc5 that form the auxiliary interface during GATOR1-stimulated GTP hydrolysis. These interactions are mediated by the Arg-1407 residue of Depdc5 and the Asp-201 and Asp-205 residues on RagC. Impairing these interactions disrupt the GAP activity of GATOR1 by causing a binding defect, leading to the blunted cellular response to low levels of amino acids. Our study uncovers a novel step in GATOR1-mediated mTORC1 inactivation that is essential for proper temporal response to nutrient scarcity.

## Experimental procedures

Chemicals and Flag-M2 affinity gel were purchased from Sigma-Aldrich. ^32^P-radioactively labeled GTP was purchased from PerkinElmer. Antibodies were purchased from the Cell Signaling Technology (CST), Abcam, Sigma-Aldrich (SA), or Millipore: Rabbit anti-HA: CST 3724; Rabbit anti-pT389-S6K1: CST 9205; Rabbit anti-S6K1: CST 2708; Rabbit anti-4EBP: CST 9452; Rabbit anti-Depdc5: Abcam ab185565; Rabbit anti-Nprl2: CST 37344; Rabbit anti-Nprl3: SA HPA011741; Rabbit anti-ULK1: CST 8054; Rabbit anti-pS757-ULK1: CST 6888; Rabbit anti-RagA: CST 4357; Rabbit anti-Raptor: Millipore 09-217; Goat-anti-rabbit HRP-linked antibody: CST 7074.

### Protein purifications

The Rag GTPase heterodimer was purified based on an established protocol (43). In brief, a pCOLADuet-1 vector encoding His_8_-Arg_10_-SUMO-tagged RagA and tagless RagC was transformed into BL21(DE3) *E. Coli* strain and was grown at 37 °C. When the optical density (OD) of the bacteria culture reached 0.8, protein expression was induced overnight with 0.5 mM IPTG at 18 °C. The next morning, the cell pellets were resuspended in resuspension buffer (50 mM NaHEPES, pH 7.4; 100 mM NaCl; 2 mM MgCl_2_; 2 mM DTT; 0.5 mM PMSF; 0.05% Triton; 100 μM GDP; and EDTA-free protease inhibitor). The resuspension was passed through a microfluidizer three times and was cleared via centrifugation. The supernatant was applied to a Ni-NTA column (Qiagen) and washed extensively. The eluate was concentrated down and passed through a MonoS column (Cytiva). The Rag-containing fractions were pooled, and the His_8_-Arg_10_-SUMO-tag was cleaved by HRV 3C protease (Pierce/ThermoFisher). Following cleavage, the protein was subjected to a second round of MonoS column to remove the cleaved tag. The Rag GTPase heterodimer was then applied to a MonoQ column (Cytiva). The eluate from the salt gradient was stripped by 20 mM EDTA, concentrated, and finally applied to a HiLoad 16/60 Superdex 200 gel-filtration column. Glycerol was added to the final, concentrated product and was flash-frozen and stored at −80 °C.

To recombinantly express GATOR1, 293-FreeStyle cells (ThermoFisher) were inoculated at 1 M/ml in SMM 293-TII serum-free culturing media (SinoBiological). After 24 h, the cells were transfected using PEI with a mixture of plasmids including Flag-Depdc5, HA-Nprl2, and HA-Nprl3. 36 h post-transfection, cells were harvested and lysed with Triton lysis buffer (TLB, 40 mM NaHEPES, pH 7.4; 100 mM NaCl; 5 mM MgCl_2_; 100 μM ATP; 10 mM Na_4_P_2_O_7_; 10 mM Na β-glycerol phosphate; 1% Triton; and EDTA-free protease inhibitor). The insoluble fractions were cleared by centrifugation, and pre-equilibrated Flag-M2 affinity gel (Sigma) was added to the lysate. GATOR1 was immunoprecipitated for 3 h at 4 °C. Flag gel was washed with TLB, followed by TLB supplemented with 300 mM NaCl. GATOR1 was then eluted with 3× Flag peptide. The eluate was concentrated down using a 100 kDa molecular-weight cut-off filter (Millipore) and passed through a HiLoad 16/60 Superdex 200 gel-filtration column to further improve the purity. The GATOR1-containing fractions were pooled, concentrated, flash frozen in the presence of 10% glycerol, and stored at −80 °C.

### GTP hydrolysis assay

Unless otherwise specified, all the biochemical assays below were carried out in Assay Buffer [50 mM NaHEPES, pH 7.4; 100 mM potassium acetate; 2 mM MgCl_2_; 2 mM DTT; and 0.1% (3-((3-cholamidopropyl)dimethylammonio)-1-propanesulfonate) (CHAPS)]. GTP hydrolysis kinetics were measured using established protocols (32, 41, 43). To measure the intrinsic hydrolysis rates of the Rag GTPase heterodimer, we used single- and multiple-turnover setups. Briefly, in a single turnover reaction, increasing concentrations of the Rag GTPases (2 nM–100 nM) were mixed with a trace amount of ^32^P-radioactively labeled GTP. Small aliquots of the reaction were taken and quenched by 0.75 M KH_2_PO_4_ (pH 3.3) at varying time points. The time points were then run on a thin layer chromatography (TLC) plate to separate the hydrolyzed GDP from the intact GTP. The reaction rates were determined and used to obtain *k*_obsd_ values, which were fitted against the concentrations of the Rag GTPases to obtain *k*_cat_ and *K*_½_ values. The multiple turnover reactions used a similar setup, except the Rag GTPases were kept constant (2 μM), while the concentration of GTP ranged from 2 μM to 100 μM. The resulting *k*_obsd_ values were corrected for the turnover number, and fitted against the concentration of GTP to get the *k*_cat_ and *K*_M_ values.

GATOR1-stimulated single-turnover GTP hydrolysis reactions were carried out by first loading 50 nM Rag-Ragulator with a trace amount of ^32^P-radioactively labeled GTP. Increasing concentrations of GATOR1 (25 nM–750 nM) were then added to the reaction to stimulate GTP hydrolysis, and the time points were analyzed accordingly. The resulting *k*_obsd_ values are fitted against the concentration of GATOR1 to get the *k*_cat_ and *K*_M_ values.

For GATOR1-stimulated multiple-turnover hydrolysis assays, 2 μM of Rag-Regulator was first incubated 100 μM of unlabeled GTP doped with a trace amount of ^32^P-radioactively labeled GTP. Increasing concentrations of GATOR1 (25 nM–750 nM) were then added to the reaction to stimulate GTP hydrolysis and the time points were analyzed accordingly. Similarly, *k*_obsd_ values were corrected for the turnover number, and fitted against the concentration of GATOR1 to obtain the *k*_cat_ and *K*_M_ values.

The assay to measure the effect of ionic strength was performed using the GATOR1-stimulated multiple-turnover hydrolysis setup, except the buffer conditions were different from the normal composition. For the low salt buffer, we used 50 mM NaHEPES, pH 7.4; 50 mM potassium acetate; 2 mM MgCl_2_; 2 mM DTT; and 0.1% CHAPS. To set up assays in increasing salt concentrations, we supplement the low salt buffer with 50, 150, and 350 mM sodium chloride. Data were acquired and analyzed similarly.

### Generation of stable cell lines

To generate Depdc5-knockout HEK-293T cell line, 2 million HEK-293T cells were seeded in a 6-well plate containing 2 ml DMEM with 10% IFS supplemented with 2 mM glutamine, penicillin (100 IU/ml), and streptomycin (100 μg/ml). The next day, the cells were transfected with guides encoded in a pX330-based CRISPR-Cas9 vector and GFP, and sorted by FACS. Single-cell clones were grown out and analyzed with Depdc5 antibody, to select the knockout line. As the CRISPR guide was transiently expressed in a plasmid, the final Depdc5-knockout HEK-293T cell line does not have puromycin resistance.

To generate stable cell lines that express wild-type Depdc5 or Depdc5(R1407D), 3 million Depdc5-knockout cells were plated in a 6-well plate containing 2 ml DMEM with 10% IFS supplemented with 2 mM glutamine, penicillin (100 IU/ml), and streptomycin (100 μg/ml). The next day, the cells were infected with lentivirus encoding HA-tagged wild-type Depdc5 or Depdc5(R1407D) and selected using puromycin 36 h post-infection. The resulting cell lines were probed with Depdc5 antibody to confirm the expression of genes of interest.

### Co-immunoprecipitation and signaling experiments

Co-immunoprecipitation and cell signaling experiments were performed based on established protocols (32, 40). Briefly, 1.5 million HEK-293T cells were plated on 10 cm petri dishes. 24 h later, the cells were treated in RPMI media containing, or deprived of, amino acids as indicated. Cells were lysed in Triton lysis buffer (TLB; 40 mM NaHEPES, pH 7.4, 5 mM MgCl_2_, 10 mM Na_4_P_2_O_7_, 10 mM Na β-glycerol phosphate, 1% Triton, and EDTA-free protease inhibitor) at specific time points and cleared by centrifugation, before proceeding with the co-immunoprecipitation. Western blots were quantified using LI-COR Odyssey imaging system.

## Data availability

All data are included in the manuscript.

## Conflict of interest

The authors declare that they have no conflicts of interest with the contents of this article.
